# Tricarbon­yl[η^5^-2-(methyl­diphenyl­phosphanium­yl)-1,3,4-triphenylcyclo­penta­dienyl]molybdenum(0)

**DOI:** 10.1107/S1600536810039747

**Published:** 2010-10-13

**Authors:** Tao Xu, Junwei Ye, Weitao Gong, Yuan Lin, Guiling Ning

**Affiliations:** a State Key Laboratory of Fine Chemicals and School of Chemical Engineering, Dalian University of Technology, Dalian 116012, People’s Republic of China

## Abstract

The title compound, [Mo(C_36_H_29_P)(CO)_3_], contains an Mo^0^ atom with a typical piano-stool coordination defined by the phospho­nium cyclo­penta­dienylide ligand η^5^-1-(methyl­diphenyl­phosphanium­yl)-2,3,5-triphenyl-2,4-cyclo­penta­dien-1-yl and by three carbonyl groups. The distance between the Mo^0^ atom and the cyclo­penta­dienyl ring is 2.0616 (13) Å.

## Related literature

For background to phospho­nium cyclo­penta­dienylides, see: Ramirez & Levy (1956 ▶); Brownie *et al.* (2007 ▶). For P—C and P=C bond lengths, see: Weast (1984 ▶) and Bart (1969 ▶), respectively.
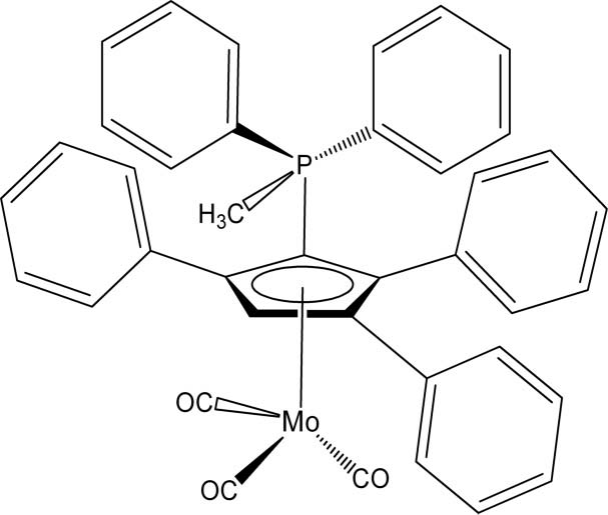


## Experimental

### 

#### Crystal data

[Mo(C_36_H_29_P)(CO)_3_]
*M* *_r_* = 672.53
Orthorhombic, 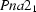

*a* = 21.609 (7) Å
*b* = 10.440 (3) Å
*c* = 14.522 (5) Å
*V* = 3276.3 (17) Å^3^
*Z* = 4
Mo *K*α radiation
μ = 0.49 mm^−1^
*T* = 293 K
0.20 × 0.18 × 0.15 mm

#### Data collection

Bruker SMART CCD area-detector diffractometer
Absorption correction: multi-scan (*SADABS*; Sheldrick, 1996 ▶) *T*_min_ = 0.909, *T*_max_ = 0.931
18060 measured reflections
7157 independent reflections
6119 reflections with *I* > 2σ(*I*)
*R*_int_ = 0.025

#### Refinement

*R*[*F* ^2^ > 2σ(*F* ^2^)] = 0.028
*wR*(*F* ^2^) = 0.053
*S* = 1.02
7157 reflections
397 parameters
1 restraint
H-atom parameters constrained
Δρ_max_ = 0.33 e Å^−3^
Δρ_min_ = −0.23 e Å^−3^
Absolute structure: Flack (1983 ▶), 3271 Friedel pairs
Flack parameter: −0.03 (2)

### 

Data collection: *SMART* (Bruker, 1997 ▶); cell refinement: *SAINT* (Bruker, 1997 ▶); data reduction: *SAINT*; program(s) used to solve structure: *SHELXS97* (Sheldrick, 2008 ▶); program(s) used to refine structure: *SHELXL97* (Sheldrick, 2008 ▶); molecular graphics: *XP* in *SHELXTL* (Sheldrick, 2008 ▶); software used to prepare material for publication: *SHELXTL*.

## Supplementary Material

Crystal structure: contains datablocks global, I. DOI: 10.1107/S1600536810039747/wm2404sup1.cif

Structure factors: contains datablocks I. DOI: 10.1107/S1600536810039747/wm2404Isup2.hkl

Additional supplementary materials:  crystallographic information; 3D view; checkCIF report

## Figures and Tables

**Table 1:** Selected bond lengths (Å)

Mo1—C39	1.927 (3)
Mo1—C38	1.937 (3)
Mo1—C37	1.946 (3)
Mo1—C4	2.374 (3)
Mo1—C1	2.379 (2)
Mo1—C5	2.387 (2)
Mo1—C3	2.417 (2)
Mo1—C2	2.419 (2)
P1—C1	1.779 (2)
P1—C24	1.793 (3)
P1—C31	1.799 (3)
P1—C25	1.806 (3)

**Fig. 1.**
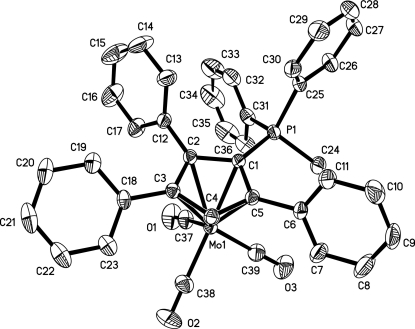
Molecular structure of the title compound showing the atom labelling and displacement ellipsoids ate the 30% probability level.

## supplementary crystallographic information

### Comment

Coordination complexes of phosphonium cyclopentadienylides have attracted more and more attention in recent years because of their application in catalysis. Although phosphonium cyclopentadienylides were first reported in 1956 by Ramirez & Levy, only few compounds beyond C_5_H_4_PPh_3_ have been reported, probably due to the difficulties in characterizing them (Brownie *et al.*, 2007). It is supposed that the behavior of this class of compounds depends on the substitutions on phosphorus.

The title compound, {Mo[η^5^-C_5_HPh_3_(PPh_2_CH_3_)](CO)_3_}, contains a Mo(0) atom in a typical piano stool coordination. The Mo atom is coordinated by a η^5^-(1,2,3,4,5-)-1-(methyldiphenylphosphonio)-2,3,5-triphenyl-2,4- cyclopentadien-1-yl ligand and three carbonyl groups. The distance between the Mo atom and the cyclopentadienyl ring is 2.0616 (13) Å. The P—C1 bond length, i.e. the phosphonium cyclopentadienylide bond, is 1.779 (2) Å, which lies between that of a typical P—C single bond (1.870 Å; Weast, 1984) and a P═C double bond (1.660 Å; Bart, 1969). This behavioutr consistent with the zwitterionic resonance structure of such phosphonium cyclopentadienylide compounds.

### Experimental

A solution of 0.49 g of C_5_HPh_3_PPh_2_CH_3_ and 1.06 g of Mo(CO)_3_(CH_3_CN)_3_ in 20 ml of THF was refluxed under argon for 3 h, during which time the solution developed a black-green color. The reaction mixture was cooled and filtered, and the solid residue was washed with THF. The resulting filtrate was then treated with 200 ml of hexane to precipitate a yellow solid that was collected and washed with hexanes. The solid was dried *in vacuo* to yield 0.40 g yellow product. X-ray quality crystals were obtained by re-crystallization from CH_2_Cl_2_ solution at 243 K by layering with hexane.

### Refinement

C-bound H atoms were placed in calculated positions (C—H = 0.93 Å) and refined in the riding-model approximation with *U*_iso_(H) = 1.2*U*_eq_(C).

### Crystal data

**Table:**

[Mo(C_36_H_29_P)(CO)_3_]	*F*(000) = 1376
*M**_r_* = 672.53	*D*_x_ = 1.363 Mg m^−^^3^
Orthorhombic, *P**n**a*2_1_	Mo *K*α radiation, λ = 0.71073 Å
Hall symbol: P 2c -2n	Cell parameters from 7157 reflections
*a* = 21.609 (7) Å	θ = 2.2–27.5°
*b* = 10.440 (3) Å	µ = 0.49 mm^−^^1^
*c* = 14.522 (5) Å	*T* = 293 K
*V* = 3276.3 (17) Å^3^	Block, yellow
*Z* = 4	0.20 × 0.18 × 0.15 mm

### Data collection

**Table:**

Bruker SMART CCD area-detector diffractometer	7157 independent reflections
Radiation source: fine-focus sealed tube	6119 reflections with *I* > 2σ(*I*)
graphite	*R*_int_ = 0.025
φ and ω scans	θ_max_ = 27.5°, θ_min_ = 2.2°
Absorption correction: multi-scan (*SADABS*; Sheldrick, 1996)	*h* = −22→28
*T*_min_ = 0.909, *T*_max_ = 0.931	*k* = −12→13
18060 measured reflections	*l* = −17→18

### Refinement

**Table:**

Refinement on *F*^2^	Secondary atom site location: difference Fourier map
Least-squares matrix: full	Hydrogen site location: inferred from neighbouring sites
*R*[*F*^2^ > 2σ(*F*^2^)] = 0.028	H-atom parameters constrained
*wR*(*F*^2^) = 0.053	*w* = 1/[σ^2^(*F*_o_^2^) + (0.0086*P*)^2^ + 1.1254*P*] where *P* = (*F*_o_^2^ + 2*F*_c_^2^)/3
*S* = 1.01	(Δ/σ)_max_ = 0.001
7157 reflections	Δρ_max_ = 0.33 e Å^−^^3^
397 parameters	Δρ_min_ = −0.23 e Å^−^^3^
1 restraint	Absolute structure: Flack (1983), 3271 Friedel pairs
Primary atom site location: structure-invariant direct methods	Flack parameter: −0.03 (2)

### Special details

**Table:**

Geometry. All e.s.d.'s (except the e.s.d. in the dihedral angle between two l.s. planes) are estimated using the full covariance matrix. The cell e.s.d.'s are taken into account individually in the estimation of e.s.d.'s in distances, angles and torsion angles; correlations between e.s.d.'s in cell parameters are only used when they are defined by crystal symmetry. An approximate (isotropic) treatment of cell e.s.d.'s is used for estimating e.s.d.'s involving l.s. planes.
Refinement. Refinement of *F*^2^ against ALL reflections. The weighted *R*-factor *wR* and goodness of fit *S* are based on *F*^2^, conventional *R*-factors *R* are based on *F*, with *F* set to zero for negative *F*^2^. The threshold expression of *F*^2^ > σ(*F*^2^) is used only for calculating *R*-factors(gt) *etc*. and is not relevant to the choice of reflections for refinement. *R*-factors based on *F*^2^ are statistically about twice as large as those based on *F*, and *R*- factors based on ALL data will be even larger.

### Fractional atomic coordinates and isotropic or equivalent isotropic displacement parameters (Å^2^)

**Table:**

	*x*	*y*	*z*	*U*_iso_*/*U*_eq_	
Mo1	0.090219 (7)	0.464808 (17)	0.09453 (2)	0.03469 (5)	
P1	−0.03449 (2)	0.68125 (5)	0.10290 (6)	0.03555 (13)	
C1	0.01081 (10)	0.5893 (2)	0.02477 (16)	0.0303 (5)	
C2	0.06846 (10)	0.6281 (2)	−0.01911 (17)	0.0331 (5)	
C3	0.09209 (10)	0.5205 (2)	−0.06697 (16)	0.0324 (5)	
C4	0.04978 (11)	0.4162 (3)	−0.05343 (19)	0.0343 (6)	
H4A	0.0528	0.3325	−0.0837	0.041*	
C5	−0.00034 (10)	0.4570 (2)	0.00062 (16)	0.0335 (5)	
C6	−0.05804 (11)	0.3791 (2)	0.01401 (19)	0.0387 (6)	
C7	−0.05867 (13)	0.2687 (3)	0.0644 (2)	0.0684 (11)	
H7A	−0.0228	0.2424	0.0942	0.082*	
C8	−0.11187 (16)	0.1949 (3)	0.0719 (3)	0.0873 (15)	
H8A	−0.1114	0.1207	0.1073	0.105*	
C9	−0.16466 (14)	0.2303 (3)	0.0279 (3)	0.0722 (10)	
H9A	−0.2002	0.1805	0.0328	0.087*	
C10	−0.16507 (14)	0.3391 (3)	−0.0234 (3)	0.0746 (11)	
H10A	−0.2011	0.3642	−0.0533	0.089*	
C11	−0.11182 (12)	0.4135 (3)	−0.0313 (2)	0.0578 (8)	
H11A	−0.1124	0.4870	−0.0675	0.069*	
C12	0.09243 (11)	0.7620 (2)	−0.02714 (18)	0.0387 (5)	
C13	0.06036 (17)	0.8457 (3)	−0.0838 (3)	0.0601 (10)	
H13A	0.0239	0.8195	−0.1122	0.072*	
C14	0.0831 (2)	0.9701 (4)	−0.0982 (4)	0.0843 (16)	
H14A	0.0616	1.0264	−0.1361	0.101*	
C15	0.1365 (2)	1.0085 (3)	−0.0566 (3)	0.1018 (15)	
H15A	0.1511	1.0913	−0.0658	0.122*	
C16	0.16889 (17)	0.9252 (3)	−0.0012 (3)	0.0841 (12)	
H16A	0.2056	0.9517	0.0263	0.101*	
C17	0.14722 (13)	0.8024 (3)	0.0138 (2)	0.0538 (7)	
H17A	0.1693	0.7467	0.0513	0.065*	
C18	0.14706 (11)	0.5116 (2)	−0.12831 (17)	0.0368 (6)	
C19	0.16267 (12)	0.6105 (3)	−0.18867 (19)	0.0490 (7)	
H19A	0.1402	0.6865	−0.1877	0.059*	
C20	0.21134 (14)	0.5965 (4)	−0.2501 (2)	0.0636 (9)	
H20A	0.2212	0.6632	−0.2900	0.076*	
C21	0.24515 (14)	0.4847 (4)	−0.2525 (2)	0.0711 (10)	
H21A	0.2777	0.4756	−0.2938	0.085*	
C22	0.23048 (14)	0.3872 (4)	−0.1937 (2)	0.0689 (10)	
H22A	0.2533	0.3118	−0.1953	0.083*	
C23	0.18213 (12)	0.3989 (3)	−0.1317 (2)	0.0514 (7)	
H23A	0.1729	0.3316	−0.0921	0.062*	
C24	−0.07093 (13)	0.5793 (3)	0.1861 (2)	0.0509 (7)	
H24A	−0.0399	0.5323	0.2192	0.076*	
H24B	−0.0980	0.5204	0.1552	0.076*	
H24C	−0.0944	0.6305	0.2285	0.076*	
C25	−0.09837 (10)	0.7619 (2)	0.04752 (19)	0.0413 (6)	
C26	−0.14111 (10)	0.8270 (2)	0.1020 (4)	0.0575 (7)	
H26A	−0.1342	0.8355	0.1649	0.069*	
C27	−0.19363 (13)	0.8787 (3)	0.0632 (3)	0.0725 (12)	
H27A	−0.2220	0.9222	0.0998	0.087*	
C28	−0.20402 (15)	0.8661 (3)	−0.0288 (3)	0.0780 (12)	
H28A	−0.2395	0.9012	−0.0548	0.094*	
C29	−0.16256 (16)	0.8021 (3)	−0.0834 (3)	0.0724 (10)	
H29A	−0.1702	0.7930	−0.1461	0.087*	
C30	−0.10906 (12)	0.7509 (3)	−0.0450 (2)	0.0497 (7)	
H30A	−0.0805	0.7090	−0.0823	0.060*	
C31	0.00960 (11)	0.7999 (3)	0.16427 (19)	0.0449 (6)	
C32	0.01231 (13)	0.9249 (3)	0.1323 (2)	0.0603 (9)	
H32A	−0.0090	0.9487	0.0794	0.072*	
C33	0.04738 (18)	1.0141 (3)	0.1807 (4)	0.0928 (14)	
H33A	0.0511	1.0975	0.1591	0.111*	
C34	0.0765 (2)	0.9780 (6)	0.2607 (5)	0.105 (2)	
H34A	0.0988	1.0387	0.2937	0.126*	
C35	0.0734 (2)	0.8571 (6)	0.2923 (3)	0.0901 (16)	
H35A	0.0941	0.8351	0.3462	0.108*	
C36	0.03997 (14)	0.7659 (4)	0.2455 (2)	0.0643 (9)	
H36A	0.0376	0.6825	0.2678	0.077*	
C37	0.14583 (13)	0.5571 (3)	0.1764 (2)	0.0522 (7)	
C38	0.15035 (11)	0.3277 (3)	0.1025 (3)	0.0553 (7)	
C39	0.05614 (15)	0.3925 (3)	0.2056 (2)	0.0515 (8)	
O1	0.17930 (11)	0.6087 (3)	0.22580 (17)	0.0856 (8)	
O2	0.18565 (10)	0.2438 (2)	0.1075 (2)	0.0902 (8)	
O3	0.03489 (12)	0.3497 (3)	0.27201 (17)	0.0861 (8)	

### Atomic displacement parameters (Å^2^)

**Table:**

	*U*^11^	*U*^22^	*U*^33^	*U*^12^	*U*^13^	*U*^23^
Mo1	0.02964 (8)	0.04116 (9)	0.03328 (9)	0.00048 (8)	−0.00279 (14)	0.00333 (15)
P1	0.0293 (3)	0.0414 (3)	0.0359 (4)	0.0018 (2)	0.0032 (4)	0.0008 (4)
C1	0.0277 (11)	0.0318 (12)	0.0314 (12)	−0.0002 (10)	−0.0014 (9)	0.0033 (10)
C2	0.0268 (11)	0.0365 (13)	0.0359 (14)	−0.0023 (10)	−0.0028 (10)	0.0038 (11)
C3	0.0282 (11)	0.0359 (12)	0.0333 (13)	0.0000 (10)	−0.0031 (10)	0.0001 (10)
C4	0.0317 (13)	0.0326 (13)	0.0386 (16)	−0.0016 (11)	−0.0025 (12)	−0.0007 (12)
C5	0.0293 (12)	0.0367 (13)	0.0345 (13)	−0.0037 (10)	−0.0031 (10)	0.0045 (11)
C6	0.0311 (13)	0.0401 (15)	0.0450 (16)	−0.0078 (11)	0.0014 (11)	0.0014 (12)
C7	0.0484 (16)	0.0592 (18)	0.098 (3)	−0.0166 (14)	−0.0147 (16)	0.0272 (17)
C8	0.079 (2)	0.068 (2)	0.115 (4)	−0.0338 (17)	−0.006 (2)	0.034 (2)
C9	0.0416 (17)	0.071 (2)	0.104 (3)	−0.0245 (17)	0.0137 (18)	−0.011 (2)
C10	0.0396 (17)	0.066 (2)	0.118 (3)	−0.0076 (16)	−0.0168 (19)	−0.011 (2)
C11	0.0389 (15)	0.0506 (17)	0.084 (2)	−0.0098 (13)	−0.0140 (15)	0.0076 (16)
C12	0.0381 (13)	0.0364 (13)	0.0417 (14)	−0.0046 (11)	0.0054 (11)	0.0000 (11)
C13	0.055 (2)	0.045 (2)	0.080 (3)	−0.0069 (16)	−0.0088 (18)	0.0147 (17)
C14	0.104 (4)	0.049 (2)	0.100 (3)	−0.010 (2)	−0.021 (3)	0.026 (2)
C15	0.124 (4)	0.051 (2)	0.131 (4)	−0.042 (2)	−0.024 (3)	0.024 (2)
C16	0.085 (3)	0.072 (2)	0.095 (3)	−0.044 (2)	−0.016 (2)	0.007 (2)
C17	0.0505 (17)	0.0516 (17)	0.0592 (19)	−0.0163 (14)	−0.0073 (14)	0.0016 (14)
C18	0.0267 (12)	0.0508 (16)	0.0329 (13)	−0.0070 (11)	−0.0016 (10)	−0.0045 (11)
C19	0.0421 (15)	0.0614 (18)	0.0435 (16)	−0.0076 (13)	0.0013 (12)	0.0002 (14)
C20	0.0468 (18)	0.100 (3)	0.0445 (18)	−0.0223 (18)	0.0086 (14)	0.0053 (17)
C21	0.0384 (16)	0.127 (3)	0.048 (2)	−0.004 (2)	0.0114 (14)	−0.015 (2)
C22	0.0472 (17)	0.099 (3)	0.061 (2)	0.0221 (18)	−0.0001 (16)	−0.024 (2)
C23	0.0430 (15)	0.0658 (19)	0.0453 (17)	0.0073 (14)	−0.0008 (13)	−0.0081 (14)
C24	0.0478 (16)	0.0606 (18)	0.0441 (17)	0.0030 (13)	0.0158 (13)	0.0102 (13)
C25	0.0262 (12)	0.0432 (14)	0.0547 (16)	−0.0006 (11)	−0.0001 (11)	0.0043 (12)
C26	0.0393 (13)	0.0641 (16)	0.0690 (19)	0.0075 (11)	0.008 (2)	0.003 (2)
C27	0.0371 (15)	0.068 (2)	0.113 (4)	0.0129 (14)	0.0129 (17)	0.012 (2)
C28	0.0386 (18)	0.084 (3)	0.112 (3)	0.0055 (16)	−0.014 (2)	0.037 (2)
C29	0.061 (2)	0.086 (3)	0.071 (2)	−0.0064 (19)	−0.0200 (18)	0.030 (2)
C30	0.0414 (15)	0.0576 (18)	0.0501 (18)	−0.0016 (13)	−0.0027 (13)	0.0150 (14)
C31	0.0347 (14)	0.0532 (17)	0.0468 (17)	0.0057 (12)	−0.0015 (12)	−0.0120 (13)
C32	0.0475 (17)	0.0502 (17)	0.083 (3)	0.0036 (13)	−0.0095 (14)	−0.0140 (15)
C33	0.074 (2)	0.053 (2)	0.152 (4)	0.0051 (18)	−0.024 (3)	−0.031 (2)
C34	0.074 (3)	0.095 (4)	0.147 (5)	0.002 (3)	−0.039 (3)	−0.061 (4)
C35	0.084 (3)	0.105 (4)	0.081 (3)	0.017 (3)	−0.031 (2)	−0.041 (3)
C36	0.064 (2)	0.074 (2)	0.055 (2)	0.0133 (17)	−0.0113 (17)	−0.0166 (17)
C37	0.0429 (16)	0.069 (2)	0.0449 (18)	−0.0062 (14)	−0.0046 (13)	−0.0003 (15)
C38	0.0469 (13)	0.0680 (16)	0.0511 (17)	0.0091 (12)	−0.0078 (19)	0.003 (2)
C39	0.0493 (18)	0.064 (2)	0.0411 (19)	0.0000 (16)	−0.0066 (15)	0.0123 (16)
O1	0.0682 (15)	0.121 (2)	0.0677 (17)	−0.0273 (15)	−0.0203 (13)	−0.0190 (15)
O2	0.0739 (13)	0.0914 (15)	0.105 (2)	0.0428 (12)	−0.016 (2)	0.007 (2)
O3	0.0861 (17)	0.118 (2)	0.0547 (15)	−0.0116 (16)	0.0013 (13)	0.0379 (15)

### Geometric parameters (Å, °)

**Table:**

Mo1—C39	1.927 (3)	C16—H16A	0.9300
Mo1—C38	1.937 (3)	C17—H17A	0.9300
Mo1—C37	1.946 (3)	C18—C19	1.396 (4)
Mo1—C4	2.374 (3)	C18—C23	1.400 (4)
Mo1—C1	2.379 (2)	C19—C20	1.387 (4)
Mo1—C5	2.387 (2)	C19—H19A	0.9300
Mo1—C3	2.417 (2)	C20—C21	1.378 (5)
Mo1—C2	2.419 (2)	C20—H20A	0.9300
P1—C1	1.779 (2)	C21—C22	1.366 (5)
P1—C24	1.793 (3)	C21—H21A	0.9300
P1—C31	1.799 (3)	C22—C23	1.384 (4)
P1—C25	1.806 (3)	C22—H22A	0.9300
C1—C5	1.446 (3)	C23—H23A	0.9300
C1—C2	1.457 (3)	C24—H24A	0.9600
C2—C3	1.417 (3)	C24—H24B	0.9600
C2—C12	1.495 (3)	C24—H24C	0.9600
C3—C4	1.435 (3)	C25—C30	1.369 (4)
C3—C18	1.488 (3)	C25—C26	1.393 (4)
C4—C5	1.404 (3)	C26—C27	1.378 (4)
C4—H4A	0.9800	C26—H26A	0.9300
C5—C6	1.501 (3)	C27—C28	1.361 (5)
C6—C7	1.365 (4)	C27—H27A	0.9300
C6—C11	1.383 (4)	C28—C29	1.371 (5)
C7—C8	1.388 (4)	C28—H28A	0.9300
C7—H7A	0.9300	C29—C30	1.390 (4)
C8—C9	1.359 (5)	C29—H29A	0.9300
C8—H8A	0.9300	C30—H30A	0.9300
C9—C10	1.358 (5)	C31—C32	1.386 (4)
C9—H9A	0.9300	C31—C36	1.396 (4)
C10—C11	1.393 (4)	C32—C33	1.391 (4)
C10—H10A	0.9300	C32—H32A	0.9300
C11—H11A	0.9300	C33—C34	1.372 (7)
C12—C13	1.386 (4)	C33—H33A	0.9300
C12—C17	1.390 (3)	C34—C35	1.345 (8)
C13—C14	1.404 (5)	C34—H34A	0.9300
C13—H13A	0.9300	C35—C36	1.375 (5)
C14—C15	1.363 (6)	C35—H35A	0.9300
C14—H14A	0.9300	C36—H36A	0.9300
C15—C16	1.377 (5)	C37—O1	1.152 (3)
C15—H15A	0.9300	C38—O2	1.164 (3)
C16—C17	1.382 (4)	C39—O3	1.158 (4)
			
C39—Mo1—C38	85.26 (16)	C13—C12—C17	119.2 (3)
C39—Mo1—C37	85.32 (13)	C13—C12—C2	117.5 (2)
C38—Mo1—C37	85.14 (13)	C17—C12—C2	123.1 (2)
C39—Mo1—C4	122.21 (11)	C12—C13—C14	119.8 (4)
C38—Mo1—C4	98.21 (14)	C12—C13—H13A	120.1
C37—Mo1—C4	152.39 (11)	C14—C13—H13A	120.1
C39—Mo1—C1	107.14 (11)	C15—C14—C13	120.2 (4)
C38—Mo1—C1	155.93 (14)	C15—C14—H14A	119.9
C37—Mo1—C1	115.78 (11)	C13—C14—H14A	119.9
C4—Mo1—C1	57.73 (8)	C14—C15—C16	120.2 (3)
C39—Mo1—C5	98.71 (11)	C14—C15—H15A	119.9
C38—Mo1—C5	123.97 (12)	C16—C15—H15A	119.9
C37—Mo1—C5	150.75 (11)	C15—C16—C17	120.4 (3)
C4—Mo1—C5	34.29 (8)	C15—C16—H16A	119.8
C1—Mo1—C5	35.32 (8)	C17—C16—H16A	119.8
C39—Mo1—C3	155.91 (11)	C16—C17—C12	120.2 (3)
C38—Mo1—C3	102.95 (14)	C16—C17—H17A	119.9
C37—Mo1—C3	117.62 (11)	C12—C17—H17A	119.9
C4—Mo1—C3	34.85 (8)	C19—C18—C23	117.9 (2)
C1—Mo1—C3	57.82 (8)	C19—C18—C3	121.5 (2)
C5—Mo1—C3	57.82 (8)	C23—C18—C3	120.4 (2)
C39—Mo1—C2	140.60 (11)	C20—C19—C18	120.6 (3)
C38—Mo1—C2	133.79 (14)	C20—C19—H19A	119.7
C37—Mo1—C2	100.83 (11)	C18—C19—H19A	119.7
C4—Mo1—C2	57.44 (9)	C21—C20—C19	120.5 (3)
C1—Mo1—C2	35.33 (7)	C21—C20—H20A	119.8
C5—Mo1—C2	58.33 (8)	C19—C20—H20A	119.8
C3—Mo1—C2	34.08 (8)	C22—C21—C20	119.5 (3)
C1—P1—C24	110.56 (12)	C22—C21—H21A	120.2
C1—P1—C31	113.33 (11)	C20—C21—H21A	120.2
C24—P1—C31	107.91 (15)	C21—C22—C23	121.1 (3)
C1—P1—C25	112.83 (13)	C21—C22—H22A	119.5
C24—P1—C25	103.97 (12)	C23—C22—H22A	119.5
C31—P1—C25	107.71 (12)	C22—C23—C18	120.4 (3)
C5—C1—C2	107.6 (2)	C22—C23—H23A	119.8
C5—C1—P1	125.34 (17)	C18—C23—H23A	119.8
C2—C1—P1	126.82 (18)	P1—C24—H24A	109.5
C5—C1—Mo1	72.61 (12)	P1—C24—H24B	109.5
C2—C1—Mo1	73.82 (13)	H24A—C24—H24B	109.5
P1—C1—Mo1	114.83 (12)	P1—C24—H24C	109.5
C3—C2—C1	107.6 (2)	H24A—C24—H24C	109.5
C3—C2—C12	125.3 (2)	H24B—C24—H24C	109.5
C1—C2—C12	126.2 (2)	C30—C25—C26	119.2 (3)
C3—C2—Mo1	72.86 (13)	C30—C25—P1	121.8 (2)
C1—C2—Mo1	70.84 (13)	C26—C25—P1	118.7 (3)
C12—C2—Mo1	130.15 (16)	C27—C26—C25	120.3 (4)
C2—C3—C4	107.8 (2)	C27—C26—H26A	119.9
C2—C3—C18	129.1 (2)	C25—C26—H26A	119.9
C4—C3—C18	122.9 (2)	C28—C27—C26	120.0 (4)
C2—C3—Mo1	73.06 (14)	C28—C27—H27A	120.0
C4—C3—Mo1	70.97 (14)	C26—C27—H27A	120.0
C18—C3—Mo1	125.42 (16)	C27—C28—C29	120.5 (3)
C5—C4—C3	109.8 (2)	C27—C28—H28A	119.8
C5—C4—Mo1	73.33 (15)	C29—C28—H28A	119.8
C3—C4—Mo1	74.18 (14)	C28—C29—C30	120.0 (3)
C5—C4—H4A	124.9	C28—C29—H29A	120.0
C3—C4—H4A	124.9	C30—C29—H29A	120.0
Mo1—C4—H4A	124.9	C25—C30—C29	120.1 (3)
C4—C5—C1	107.3 (2)	C25—C30—H30A	120.0
C4—C5—C6	123.3 (2)	C29—C30—H30A	120.0
C1—C5—C6	128.7 (2)	C32—C31—C36	120.1 (3)
C4—C5—Mo1	72.38 (14)	C32—C31—P1	120.3 (2)
C1—C5—Mo1	72.07 (12)	C36—C31—P1	119.5 (2)
C6—C5—Mo1	128.72 (16)	C31—C32—C33	119.0 (3)
C7—C6—C11	117.8 (2)	C31—C32—H32A	120.5
C7—C6—C5	122.4 (2)	C33—C32—H32A	120.5
C11—C6—C5	119.7 (2)	C34—C33—C32	119.6 (4)
C6—C7—C8	121.2 (3)	C34—C33—H33A	120.2
C6—C7—H7A	119.4	C32—C33—H33A	120.2
C8—C7—H7A	119.4	C35—C34—C33	121.6 (4)
C9—C8—C7	120.5 (3)	C35—C34—H34A	119.2
C9—C8—H8A	119.8	C33—C34—H34A	119.2
C7—C8—H8A	119.8	C34—C35—C36	120.5 (5)
C10—C9—C8	119.4 (3)	C34—C35—H35A	119.8
C10—C9—H9A	120.3	C36—C35—H35A	119.8
C8—C9—H9A	120.3	C35—C36—C31	119.2 (4)
C9—C10—C11	120.4 (3)	C35—C36—H36A	120.4
C9—C10—H10A	119.8	C31—C36—H36A	120.4
C11—C10—H10A	119.8	O1—C37—Mo1	178.2 (3)
C6—C11—C10	120.7 (3)	O2—C38—Mo1	178.8 (3)
C6—C11—H11A	119.7	O3—C39—Mo1	179.1 (3)
C10—C11—H11A	119.7		
